# Identification of Novel Microsatellite Markers Flanking the *SMN1* and *SMN2* Duplicated Region and Inclusion Into a Single-Tube Tridecaplex Panel for Haplotype-Based Preimplantation Genetic Testing of Spinal Muscular Atrophy

**DOI:** 10.3389/fgene.2019.01105

**Published:** 2019-11-06

**Authors:** Mingjue Zhao, Mulias Lian, Felicia S.H. Cheah, Arnold S.C. Tan, Anupriya Agarwal, Samuel S. Chong

**Affiliations:** ^1^Department of Pediatrics, Yong Loo Lin School of Medicine, National University of Singapore, Singapore, Singapore; ^2^Preimplantation Genetic Diagnosis Center, Khoo Teck Puat—National University Children’s Medical Institute, National University Health System, Singapore, Singapore; ^3^Clinic for Human Reproduction, Department of Obstetrics and Gynecology, National University Hospital, Singapore, Singapore; ^4^Molecular Diagnosis Center and Clinical Cytogenetics Service, Department of Laboratory Medicine, National University Hospital, Singapore, Singapore

**Keywords:** microsatellite, preimplantation genetic testing for monogenic disorders (PGT-M), spinal muscular atrophy (SMA), spinal motor neuron (SMN), multiplex PCR, haplotype

## Abstract

Preimplantation genetic testing for the monogenic disorder (PGT-M) spinal muscular atrophy (SMA) is significantly improved by supplementation of *SMN1* deletion detection with marker-based linkage analysis. To expand the availability of informative markers for PGT-M of SMA, we identified novel non-duplicated and highly polymorphic microsatellite markers closely flanking the *SMN1* and *SMN2* duplicated region. Six of the novel markers within 0.5 Mb of the 1.7 Mb duplicated region containing *SMN1* and *SMN2* (*SMA6863, SMA6873, SMA6877, SMA7093, SMA7115*, and *SMA7120*) and seven established markers (*D5S1417, D5S1413, D5S1370, D5S1408, D5S610, D5S1999*, and *D5S637*), all with predicted high heterozygosity values, were selected and optimized in a tridecaplex PCR panel, and their polymorphism indices were determined in two populations. Observed marker heterozygosities in the Chinese and Caucasian populations ranged from 0.54 to 0.86, and 98.4% of genotyped individuals (185 of 188) were heterozygous for ≥2 markers on either side of *SMN1*. The marker panel was evaluated for disease haplotype phasing using single cells from two parent–child trios after whole-genome amplification, and applied to a clinical IVF (*in vitro* fertilization) PGT-M cycle in an at-risk couple, in parallel with *SMN1* deletion detection. Both direct and indirect test methods determined that none of five tested embryos were at risk for SMA, with haplotype analysis further identifying one embryo as unaffected and four as carriers. Fresh transfer of the unaffected embryo did not lead to implantation, but subsequent frozen-thaw transfer of a carrier embryo produced a pregnancy, with fetal genotype confirmed by amniocentesis, and a live birth at term.

## Introduction

Spinal muscular atrophy (SMA; type I, OMIM# 253300; type II, OMIM# 253550; type III, OMIM# 253400; type IV, OMIM# 271150) is a severe to lethal disorder characterized by progressive muscle weakness resulting from degeneration and loss of anterior horn cells in the spinal cord and brain stem nuclei (Prior and Russman, 2000 Feb 24 [last update: December 22, 2016]). With an incidence of 1 in 10,000 live births and a carrier frequency of 1 in 40–60, it is the second most common fatal autosomal recessive disorder and the number one genetic cause of infant death (Prior et al., 2008; Gitlin et al., 2010). The gene responsible for SMA is *survival motor neuron 1* (*SMN1*), located in a ∼1.7 Mb tandemly duplicated region on chromosome 5q13.2 which also contains a duplicate gene *SMN2*. Homozygous loss of *SMN1* due to deletion and/or conversion to *SMN2* causes ∼95% of SMA cases, with the remaining 5% caused by compound heterozygosity of an *SMN1* deletion and an *SMN1* intragenic mutation (Wirth, 2000; D’Amico et al., 2011). *SMN2* deletions are not pathogenic, but increased *SMN2* copies lessen SMA disease severity (Feldkotter et al., 2002). SMA type I, the most severe form accounting for ∼50% of SMA patients, is lethal before age 2 (Prior and Russman, 2000 Feb 24 [last update: December 22, 2016]; Meldrum et al., 2007). A recently approved drug *nusinersen* and a gene therapy *onasemnogene abeparvovec* have been demonstrated to be effective in improving motor function and life span in SMA patients, although their high cost may be an issue for some families (Hoy, 2017; Hoy, 2019). Given the severity of the disease, the high carrier frequency, and the current lack of a cost-effective cure, proper family planning and prevention through prenatal diagnosis or preimplantation genetic testing for monogenic disorder (PGT-M) represents the best choice for at-risk couples.

PGT-M of SMA by *SMN1* deletion analysis is challenging because *SMN1* and *SMN2* differ at only five nucleotide positions and because large regions surrounding each gene share significant sequence identity. Most SMA PGT-M assays interrogate a single nucleotide difference between *SMN1* and *SMN2* at position c.840 in exon 7, in order to determine homozygous deletion of *SMN1*. As these assays only detect presence or absence of *SMN1*, unaffected embryos cannot be differentiated from carrier embryos. Published methods for determining *SMN1* and/or *SMN2* copy number from genomic samples are robust (Anhuf et al., 2003; Huang et al., 2007; Stabley et al., 2015; Feng et al., 2017) but have yet to be shown to work reliably from single cells, either directly or after whole-genome amplification (WGA).

The current *SMN1* direct detection assays are also vulnerable to misdiagnosis due to allele dropout (ADO) (Fiorentino et al., 2003) and/or DNA contamination, and at least one affected birth resulting from possible false-negative diagnosis has been documented by the European Society of Human Reproduction and Embryology PGD Consortium (Wilton et al., 2009).

In addition, due to the presence of *SMN2*, direct detection of non-deletional mutations in *SMN1* is unreliable with any of the reported assays, thus making indirect mutation detection by linkage analysis the best option for at-risk couples carrying such mutations. Linked polymorphic markers have been used in SMA PGT-M, usually to supplement direct mutation detection and to simultaneously monitor for ADO and DNA contamination, thus increasing diagnostic confidence. Markers within the duplicated segment are also duplicated and can complicate haplotype analysis. Those outside the duplicated segment are useful for PGT-M, but to date, only eight such single-copy markers have been reported (Moutou et al., 2003; Moutou et al., 2006; Girardet et al., 2008; Burlet et al., 2010; Korzebor et al., 2013). Some markers are located >5 Mb away from *SMN1*, increasing the possibility of misdiagnosis due to marker-mutation recombination. Although bi-allelic single nucleotide polymorphism (SNP)–based karyomapping has been used in linkage analysis for PGT-M of SMA (Konstantinidis et al., 2015), the SNP coverage at the *SMN1* locus is relatively low, thus reducing reliability of this method for SMA PGT-M.

Taken together, current SMA PGT-M tests suffer from a combination of sub-optimal reliability in direct *SMN1* deletion detection and scarcity of closely linked polymorphic markers for indirect linkage analysis. To increase the availability of multi-allelic informative linked markers for SMA PGT-M, we searched for novel non-duplicated microsatellite markers adjacent to the *SMN1* and *SMN2* duplicated region that were highly polymorphic. We then combined these novel markers with previously established/published markers that were also highly polymorphic into a single-tube tridecaplex PCR panel. The utility of this marker panel in establishing disease haplotype phase was demonstrated in whole-genome amplified single cells from cell lines of two parent–child trios, and subsequently applied to clinical IVF PGT-M of SMA in an at-risk couple, in parallel with direct *SMN1* deletion analysis.

## Materials and Methods

### Biological Samples and Single-Cell Processing

Genomic DNA was extracted from 40 cell lines purchased from Coriell Cell Repositories (CCR, New Jersey, USA) and from 92 unrelated and anonymized cord bloods of Chinese babies born at the National University Hospital. The Caucasian Human Variation DNA panel (HD100CAU) was purchased from CCR. Single cells were isolated from GM03620, GM06581, and six other CCR cell lines derived from two SMA parent–child trios. The first trio of cell lines comprise GM03813 (affected child) and GM03814 and GM03815 (his carrier parents), while the second trio comprise GM23686 (affected child) and GM23687 and GM23688 (her carrier parents). Single-cell isolation, processing, and WGA have been described elsewhere (Chen et al., 2015). Briefly, isolated single cells in 2 µl of 1× phosphate-buffered saline (PBS, Cell Signaling Technology, Massachusetts, USA) were lysed by adding 1.5 µl of 0.6 M potassium hydroxide (KOH, Sigma-Aldrich, Missouri, USA), heated at 65°C for 10 min, rapidly cooled to 4°C, and neutralized by the addition of 1.5 µl of 0.6 M Tricine (Sigma-Aldrich). The lysed cells were subjected to WGA using illustra GenomiPhi^™^ V2 DNA Amplification Kit (GE Healthcare, Illinois, USA) according to manufacturer’s instructions, except that the incubation time was 4 h. This study was approved by the Institutional Review Board of the National University of Singapore (07-123E and 13-309E).

### Microsatellite Marker Identification, Selection, and Genotyping

Approximately 0.5 Mb of DNA sequences upstream and downstream of the chromosome 5q13.2 duplicated region were downloaded from the UCSC genome browser (GRCh37/hg19 assembly). The strategy for identification and selection of markers and for primer design have been described elsewhere (Chen et al., 2015; Zhao et al., 2017). Briefly, di-, tri-, tetra-, and penta- nucleotide repeats with >80% sequence match and with alignment scores of >54, 80, 66, and 52, respectively, were selected. One primer of each pair was tailed at the 5’ end with one of three bacteriophage M13 sequences (**Table 1**). Three additional primers, each consisting of one of the three M13 sequences, were labeled at the 5’ end with 6-Fam, Hex, or Ned.

**Table 1:** Tridecaplex microsatellite marker PCR details.

Microsatellite marker	Repeat motif	PCR primer sequence (5’- > 3’)^†^	Concentration (µM)	Amplicon size (bp)‡	He‡		Ho‡	
					CH	CAU	CH	CAU
*D5S1417*	(TG)_n_	F ^M13-2^GAGACATTCAACTCAGCTAGAGAG	0.2	206–228	0.69	0.75	0.63	0.7
		R ^¶^CCCTGGAAACACTGCAATCCCTC						
*D5S1413*	(GT)_n_	F ^M13-1^ TGGCTACAGGCCAGATGAG	0.1	143–167	0.62	0.56	0.63	0.59
		R ^¶^GAAAATAGGCTTGTGAAACCAACGC						
***SMA6863***	(GA)_n_	F ^M13-1^GGCCTCCTTAAACTAGCTGTTATG	0.3	348–384	0.81	0.71	0.84	0.69
		R ^¶^ACTGCCTCTACCTCTGAACCTC						
***SMA6873***	(AC)_n_	F ^M13-1^CTAAATGTCGGTCTGGCTGTG	0.2	295–327	0.82	0.76	0.77	0.78
		R GATTGAAACAAAGACACCTAACTTCTCAGG						
*D5S1370*	(TG)_n_	F ^M13-2^GAGCCATATTTGAAACCCAAGCC	0.2	177–193	0.68	0.52	0.68	0.57
		R ^¶^AGGCCCATTCACTTGCAGAC						
***SMA6877***	(TG)_n_	F ^M13-3^TCTGAGTCAAAGCACTGAGTTTCC	0.4	271–293	0.81	0.79	0.83	0.8
		R CTTGGACCCAGGTTGTTAGAG						
*SMN2*								
*SMN1*								
*D5S1408*	(AC)_n_	F ^M13-1^TGTAGAGATGCTTCTGTGGCTC	0.1	246–274	0.76	0.72	0.79	0.7
		R ^ψ^TTAGCAGAGGCAGGGTTTCACC						
***SMA7093***	(TG)_n_	F ^M13-1^ACTAGATGCCTCAGCAACCAG	0.1	178–206	0.57	0.61	0.57	0.56
		R AGTGCTCCAGATGGTTCGTC						
*D5S610*	(TG)_n_	F ^M13-2^ATCTTTTGTTAAGCTCCTCCAGTG	0.2	143–175	0.84	0.8	0.8	0.8
		R ^¶^CATGCCCAGCCTAAACTGAAC						
***SMA7115***	(AG)_n_	F ^M13-2^GGAGAACTTTCAAGAGCTAGAGG	0.2	281–311	0.81	0.84	0.85	0.81
		R CCAGGATGTATAAAGAAGATGGTCTG						
***SMA7120***	(AC)_n_	F ^M13-3^CACCACATCCAAGATCTGTGG	0.2	307–325	0.75	0.79	0.75	0.75
		R GGGTATAAAATCCTGGGCTAACAGC						
*D5S1999*	(GA)n	F ^M13-2^AATCTCCTGGCAACAGTGATCTC	0.3	319–327	0.62	0.52	0.62	0.6
		R ^¶^CAACTCAGAAATTGATGGGACACAG						
*D5S637*	(CA)n(CT)n	F ^M13-1^CACGAGGTGCTTCACCACC	0.1	235–243	0.52	0.74	0.54	0.86
		R ^¶^CCAATAATATGGCAGGTTTATGAGCTG						

Markers highlighted in bold are novel.

^†^Primer sequences were based on genome assembly build GRCh37/hg19.

^¶^5’ GTTT tailed; ^ψ^ 5’ GT tailed.

^M13-1^, 5’ GGTTTTCCCAGTCACGAC tailed; ^M13-2^, 5’ GTAAAACGACGGCCAGTG tailed; ^M13-3^, 5’ CATGGTCATAGCTGTTTCCTG tailed.

***^‡^*** Amplicon size, H_e_ (expected heterozygosity), and H_o_ (observed heterozygosity) were determined from 92 CH (Chinese) and 96 CAU (Caucasian) DNA samples.

Microsatellite markers were individually genotyped against an initial panel of 32 cell line DNAs to exclude those with low heterozygosity values and complex electrophoretic peak patterns. Markers with high heterozygosity values were selected for co-amplification in a tridecaplex PCR reaction. Multiplex PCR was performed in a 20 µl reaction consisting of 10 ng genomic DNA or 1 µl of single-cell WGA product, 1× QIAGEN Multiplex Mastermix (Qiagen, Hilden, Germany), 0.5× Q-Solution (Qiagen), and 0.1–0.4 µM of primers for each marker. Thermal cycling involved a 15 min denaturation/activation at 95°C, followed by 30 cycles of 98°C for 45 s, 65°C for 90 s, and 72°C for 60 s, and ended with a 30 min incubation at 60°C. For product visualization, a 2 µl aliquot of multiplex PCR product was used as template for extension labeling in a 20 µl reaction using 0.2 µM each of 6-Fam–labeled M13-1 primer, Hex-labeled M13-2 primer, and Ned-labeled M13-3 primer. Thermal cycling was identical to the multiplex PCR, except that 10 cycles were performed. The optimized tridecaplex marker panel was used to genotype 92 Chinese and 96 Caucasian DNA samples to determine heterozygosity values for each marker. It was also tested on WGA product from 16 single cells of GM03620 and GM06581 to assess its utility and validity for application to PGT-M of SMA following WGA.

A 1 µl aliquot of extension-labeled multiplex PCR product was mixed with 9 µl of Hi-Di^™^ Formamide (Applied Biosystems, California, USA) and 0.3 µl of GeneScan^™^ 500 ROX^™^ dye size standard (Applied Biosystems). Mixtures were denatured at 95°C for 5 min, cooled to 4°C, and resolved in a 3130xl Genetic Analyzer (Applied Biosystems) using a 36 cm capillary filled with POP-7^™^ polymer (Applied Biosystems). Samples were electrokinetically injected at 1.2 kV for 23 s and electrophoresed for 20 min at 60°C under GeneScan^™^ application. Data analysis was performed as described elsewhere (Zhao et al., 2016).

### Haplotype Phasing of Maternal and Paternal Mutant and Wild-Type Alleles

The tridecaplex marker panel was validated on CCR cell lines of two carrier parent-affected child trios. For each marker, both alleles present in the affected offspring are assumed to be linked to the maternal and paternal *SMN1*-deleted chromosomes, and assigned accordingly. Consequently, the other allele present in each parent must be linked to their wild-type chromosomes. The assigned alleles of all 13 markers will generate a distinct haplotype that uniquely identifies each maternal and paternal mutant and wild-type chromosome.

### *SMN1* Deletion Analysis

**Figure 1:**
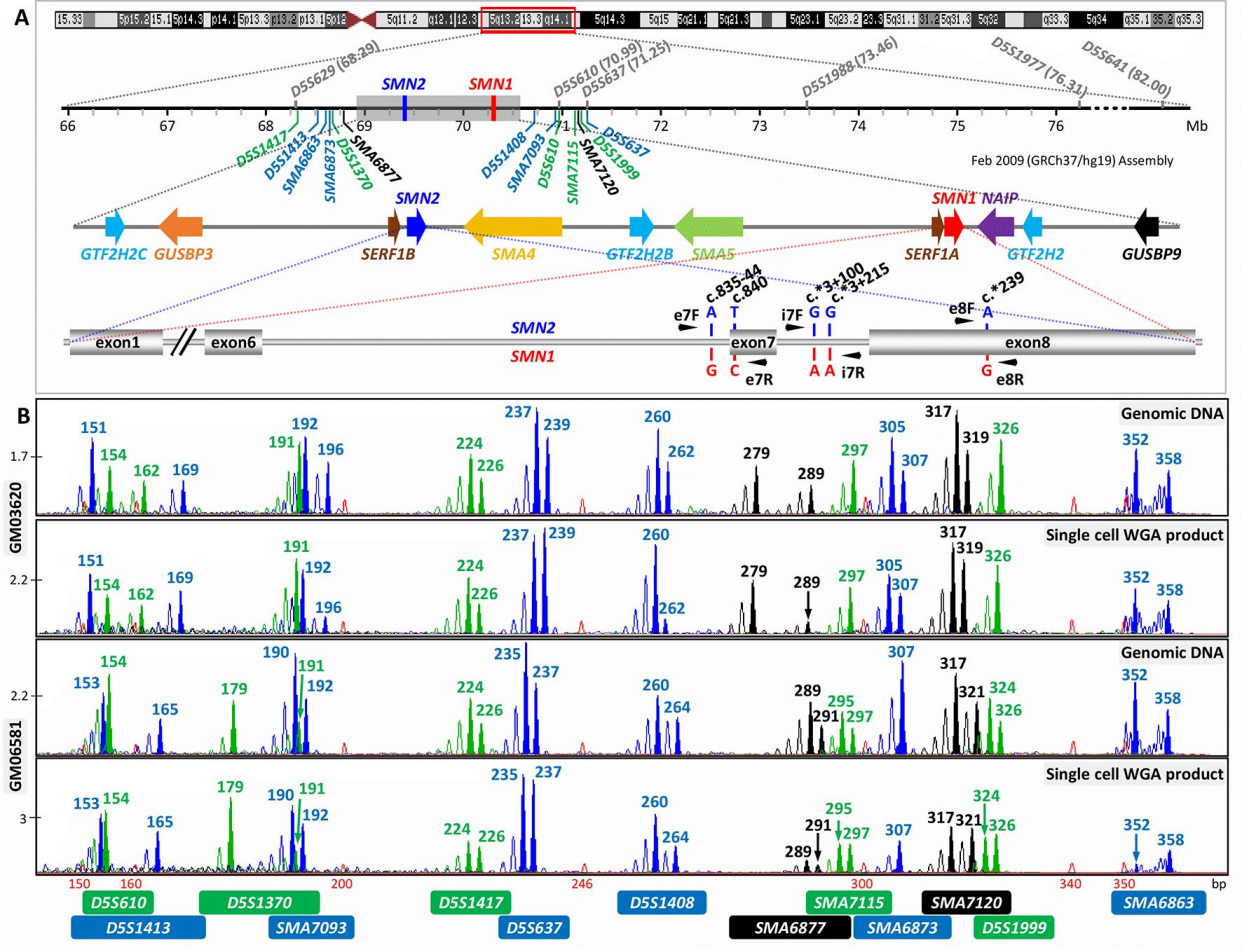
Strategy for combined indirect and direct preimplantation genetic testing (PGT) of spinal muscular atrophy. **(A)** Schematic illustration of chromosome 5q13.2 showing the ∼1.7 Mb *SMN1* and *SMN2* duplicated region, the 13 multiplex PCR markers (in blue, black, and green) flanking the duplicated region, and the five nucleotide differences between *SMN1* and *SMN2*. Black arrowheads indicate positions of primers designed to amplify segments of exon 7, intron 7, and exon 8. Previously reported markers used in PGT are in grey. **(B)** Electropherograms after multiplex PCR of genomic DNA and single-cell whole-genome amplification (WGA) product from two selected cell lines.

Primers were designed to amplify three *SMN* gene fragments known to contain single nucleotide differences between *SMN1* and *SMN2* (**Figure 1A** and **Table 2**). Triplex PCR amplification was performed in a 20 µl reaction containing 10 ng genomic DNA or 1 µl of single-cell WGA product, 1× QIAGEN Multiplex Mastermix, and 0.2 µM of each primer. Thermal cycling involved a 15 min incubation at 95°C, followed by 30 cycles of 98°C for 45 s, 65°C for 90 s, and 72°C for 60 s, and ended with a 60°C incubation for 30 min. A 2.5 µl aliquot of the amplification product was subjected to PCR cleanup by adding 10 U exonuclease I (Exo I, GE Healthcare) and 1 U Shrimp Alkaline Phosphatase (SAP, GE Healthcare) in a final volume of 4.5 µl, incubated at 37°C for 45 min, and heat-inactivated at 80°C for 15 min. Triplex minisequencing was performed in a 10 µl reaction consisting of 0.4 µM of each minisequencing primer, 2.5 µl of SNaPshot^®^ Multiplex Reaction Mix (Applied Biosystems), and 4.5 µl of cleanup product. Each minisequencing primer contained additional non-specific nucleotides of different length at its 5’ end to improve electrophoretic separation among the three groups of minisequencing products. Thermal cycling involved 30 cycles of 96°C for 10 s, 50°C for 5 s, and 60°C for 30 s. One unit of SAP was added to the triplex minisequencing reaction, incubated at 37°C for 1 h, and heat-inactivated at 75°C for 15 min.

**Table 2:** Triplex *SMN* amplicon PCR and minisequencing primers and expected products.

Triplex PCR	Amplicon	Forward and reverse PCR primer sequence (5’- > 3’)^†^	Concentration (µM)	Amplicon size (bp)
Triplex minisequencing	Nucleotide difference: *SMN1/SMN2*	Minisequencing primer position^†^	Concentration (µM)	Extended primer length*
	exon7	For: ACAAAATGCTTTTTAACATCCATATAAAGCTATC; Rev: CATAATGCTGGCAGACTTACTCC	0.2; 0.2	169
	intron7	For: GTGAATCTTACTTTTGTAAAACTTTATGGTTTG; Rev: GATATAAAATGGCATCATATCCTAAAGCTC	0.2; 0.2	250
	exon8	For: GTGGAATGGGTAACTCTTCTTG; Rev: ACTGCCTCACCACCGTG	0.2; 0.2	139
	exon7 c.840: C/T	For: ^T1^CTTTATTTTCCTTACAGGGTTT	0.4	*SMN1*/C: 43; *SMN2*/T: 44
	intron7 c.*3+215: A/G	Rev: ^T2^TGAAAGTATGTTTCTTCCACA	0.4	*SMN1*/T: 39; *SMN2*/C: 41
	exon8 c.*239: G/A	Rev: ^T2^TGGCCTCCCACCCCCACC	0.4	*SMN1*/C: 34; *SMN2*/T: 37

^†^Primer sequences were based on genome assembly build GRCh37/hg19.

^T1^, GACTGACTGACT was added to the 5’ of the primer; ^T2^, CTGACTGACT was added to the 5’ of the primer.

*Apparent nucleotide length of the C- or T-extended primer based on its electrophoretic migration.

A 1 µl aliquot of the final minisequencing product was mixed with 9 µl of Hi-Di^™^ Formamide and 0.1 µl of GeneScan^™^ 120 LIZ^™^ dye size standard (Applied Biosystems). Mixtures were denatured at 95°C for 5 min, cooled to 4°C, and resolved in a 3130xl Genetic Analyzer using a 36 cm capillary filled with POP-7^™^ polymer. Samples were electrokinetically injected at 1.2 kV for 23 s and electrophoresed for 20 min at 60°C under the SNaPshot application.

### IVF PGT-M Case

The optimized and validated tridecaplex microsatellite marker PCR assay was applied clinically to an SMA at-risk couple. Written informed consent was obtained from the couple for the IVF PGT-M and for the presentation of their clinical case for publication.

*SMN1* deletion analysis by triplex *SMN* fragment PCR and minisequencing was first performed on genomic DNA from the couple and their affected son to confirm the homozygous absence of *SMN1* in the affected son. Single-tube tridecaplex microsatellite PCR was performed on a separate aliquot of genomic DNAs to identify informative markers necessary to establish the maternal and paternal mutant/*SMN1*-deleted and normal/*SMN1*-positive haplotypes.

All embryos were generated by intracytoplasmic sperm injection (ICSI) of oocytes. One to two blastomeres were biopsied from each embryo on day three and analyzed separately. Each blastomere was lysed by adding 1.5 µl of 0.6 M KOH, heated at 65°C for 10 min, rapidly cooled to 4°C, and neutralized with 1.5 µl of 0.6 M Tricine. Lysed blastomere samples were subjected to WGA using the illustra GenomiPhi^™^ V2 DNA Amplification Kit according to manufacturer’s instructions, except that the incubation time was 4 h, and separate 2 µl aliquots of WGA product were used for the triplex *SMN* fragment PCR–minisequencing and tridecaplex microsatellite marker PCR assays, as described above, in 50 µl reactions.

## Results

### Identification of Novel Microsatellite Markers and Development of a Tridecaplex PCR Marker Panel

Forty-five and 39 microsatellite markers located ≤0.5 Mb upstream and downstream of the ∼1.7 Mb *SMN1* and *SMN2* duplicated region, respectively, were identified by *in silico* mining, of which a total of 51 markers satisfied the selection criteria (**Table S1**). The percentage of matches refers to the extent of perfect repeats within a tandem repeat, whereas the alignment score is a weighting for match, mismatch, and indels, and reflects how long and perfect the repeat is. Markers with higher scores indicate a likely higher polymorphism. Thirty-two markers were excluded due to difficulty in designing specific primer pairs because of their location within either *Alu* or other repeat sequences. Although slightly >0.5 Mb downstream of the 5q13.2 duplicated region and not captured in the initial search, marker *D5S637* was included with the remaining 19 markers for experimental evaluation because it was previously assessed to be highly polymorphic in the Caucasian population (Korzebor et al., 2013). The 20 markers were individually screened against 32 random DNA samples. Four markers displayed complex peak patterns (*SMA6870*, *SMA6874.3*, *SMA7092*, and *SMA7103.2*), while another two had low heterozygosity values (*D5S1364* and *SMA7101.2*), and these markers were dropped from further consideration.

When the remaining 14 markers were combined into a multiplex PCR reaction, marker *D5S629* was observed to interfere with *D5S1408.* Although *D5S629* is highly polymorphic and has been used in SMA PGT-M previously, one of its primers anneals within an *Alu* repeat. Given the close proximity of *D5S1408* to *SMN1*, it was retained in the panel while *D5S629* was dropped. The final optimized panel of 13 markers includes 7 established/published markers (*D5S1417*, *D5S1413*, *D5S1370*, *D5S1408*, *D5S610*, *D5S1999*, and *D5S637*) and 6 novel markers (*SMA6863*, *SMA6873*, *SMA6877*, *SMA7093*, *SMA7115*, and *SMA7120*). As shown in **Figure 1A**, the six upstream and seven downstream markers lie within 2 Mb upstream and 1 Mb downstream, respectively, of *SMN1*. After amplification from multiplex PCR, each marker can be easily distinguished by its allele size range, peak pattern, and peak color after capillary electrophoresis (**Figure 1B**).

The marker panel was validated on WGA product of 16 single cells isolated from cell lines GM03620 and GM06581 to evaluate its utility in SMA PGT-M following WGA. All 13 markers amplified successfully from 13 out of 16 single-cell WGA products (**Figure 1B**), with the 14^th^ and 15^th^ cells failing to detect *D5S1408* and *SMA6877*, respectively. The last cell had amplification failures of *D5S1370*, *SMA7093*, *SMA6877*, *SMA6873*, and *D5S1999*, which was highly unusual and possibly related to the biological condition of the cell. Among the 13 cells where all 13 markers amplified successfully, ADO was not observed for any marker in 6 cells, while the remaining 7 cells exhibited ADO of 1 to 4 individual markers. Individual marker ADO rates were calculated from all cells where amplification of that marker was successful, and ranged from 0% to 37.5% (data not shown), which was within expectations for analysis from single-cell WGA product (Handyside et al., 2004; Spits et al., 2006; Renwick et al., 2007; Renwick et al., 2010).

### Marker Polymorphism Evaluation

**Figure 2:**
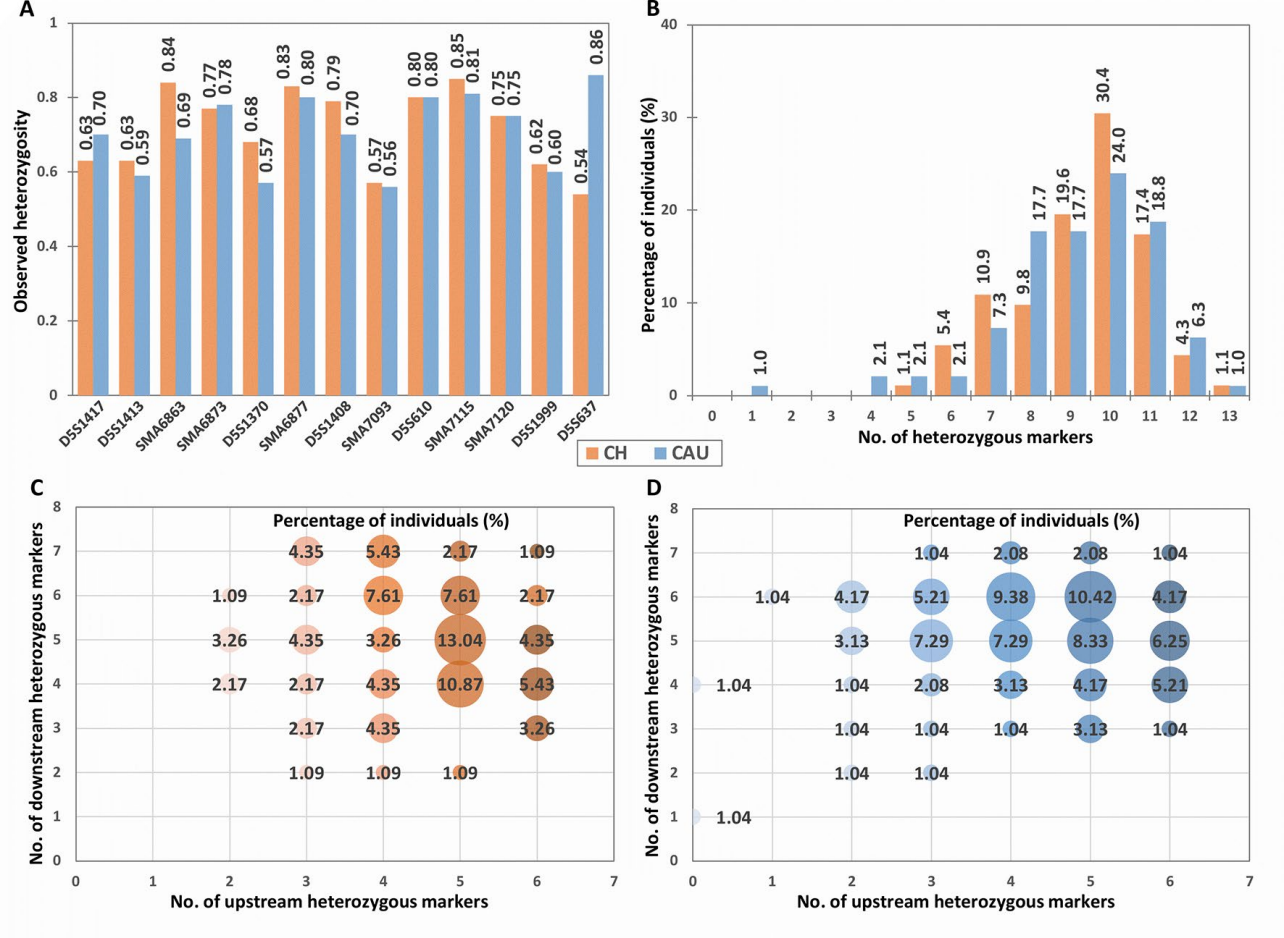
Population heterozygosity analysis of the tridecaplex panel markers. **(A)** Observed heterozygosity of each marker. **(B)** Percentage of individuals heterozygous for different numbers of markers. **(C)** Percentage of Chinese heterozygous for different numbers of markers upstream and downstream of the *SMN1* and *SMN2* duplicated region. **(D)** Percentage of Caucasians heterozygous for different numbers of markers upstream and downstream of the *SMN1* and *SMN2* duplicated region. CH, Chinese; CAU, Caucasian.

To assess the expected and observed heterozygosities (H_e_ and H_o_) of each marker, we genotyped 92 Chinese and 96 Caucasian DNA samples using the tridecaplex PCR marker panel. Between 5 and 17 alleles were observed for each marker (**Figure S1** and **Table S2**), with H_e_ and H_o_ ranges of 0.52–0.84 and 0.54–0.85, respectively, in the Chinese and 0.52–0.84 and 0.56–0.86, respectively, in Caucasians (**Tables 1** and **S2**, and **Figure 2**). *SMA7115* was the most polymorphic in the Chinese, with an H_o_ of 0.85, whereas *D5S637* was the most polymorphic in Caucasians, with an H_o_ of 0.86.

Differences in marker heterozygosity were observed between the two population groups. For example, the H_o_ of *D5S637* was much higher in Caucasians (0.86) compared to the Chinese (0.54). Nevertheless, 99.5% of each population sample was heterozygous for at least four panel markers (**Figure 2**). In addition, all Chinese samples and 96.9% of Caucasian samples were heterozygous for two or more markers upstream as well as downstream of *SMN1* (**Figures 2C, D**). Furthermore, individuals with a total of 10 heterozygous markers and individuals with 5 to 6 heterozygous markers at each end of *SMN1* comprise the largest proportions in both population groups. These observations suggest that the tridecaplex marker panel contains sufficient marker redundancy to be informative for most SMA PGT-M cases without the need to identify additional markers or to develop case-specific customized marker panels.

### Evaluation of Tridecaplex Marker Panel for Disease Haplotype Phasing

Tridecaplex marker PCR for genotyping and multi-marker haplotyping was performed in parallel with direct *SMN1* deletion analysis on whole-genome amplified single cells isolated from cell lines of SMA-affected children (GM03813 and GM23686) and their respective carrier parents (GM03814 and GM03815, and GM23687 and GM23688). The *SMN1* deletion analysis involved multiplex PCR and multiplex minisequencing of three *SMN* amplicons to maximize sensitivity and specificity for *SMN1* detection. The indirect linkage test and direct mutation detection test were validated on separate aliquots of WGA product from the same cells.

We initially validated the assays using 8 replicate single cells of each cell line (total of 24 cells) of the first trio. One cell from GM03815 failed to produce any amplification product from either of the two tests, suggesting that the initial WGA had failed, and was excluded, leaving 23 cells available for analysis.

**Figure 3:**
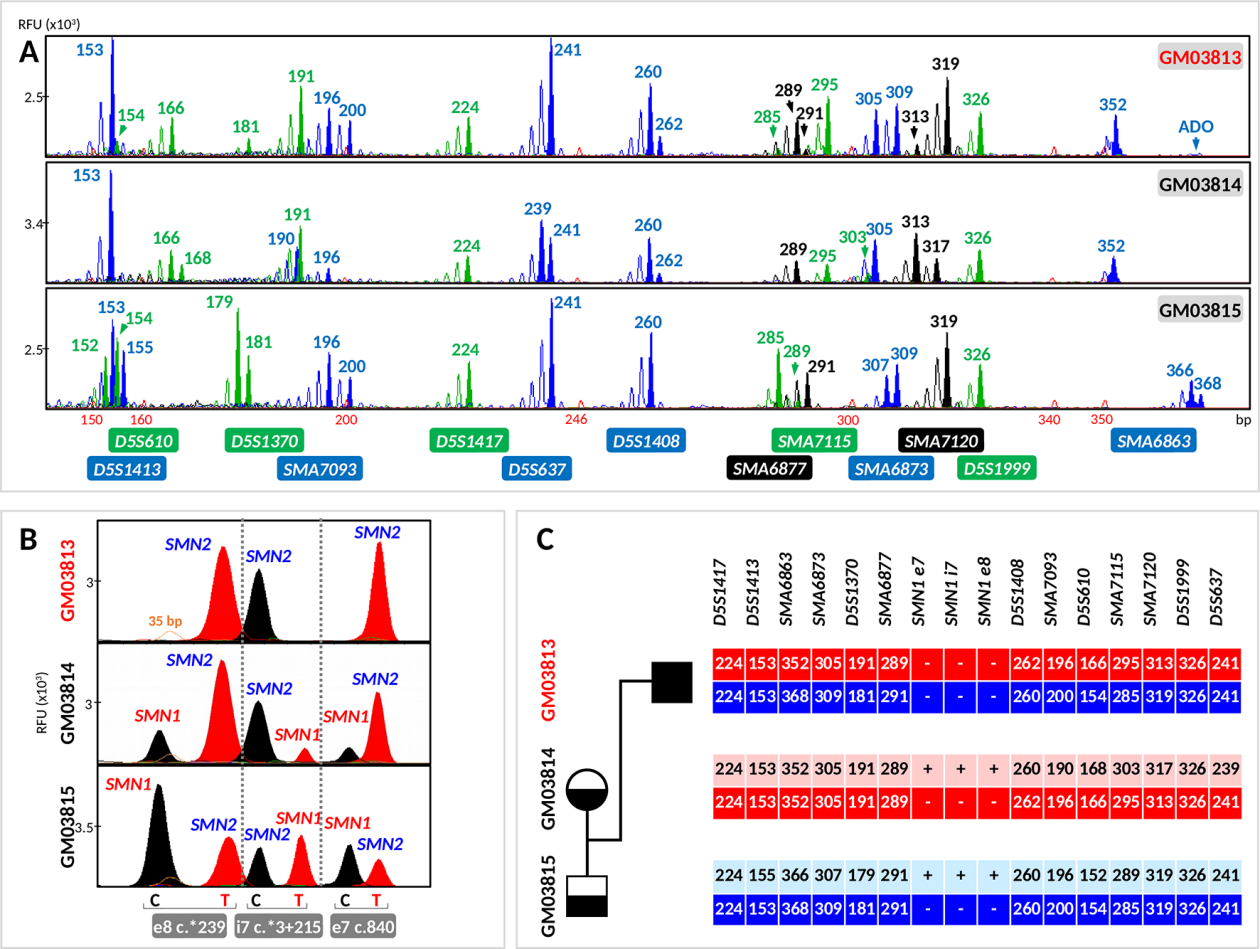
Evaluation of the tridecaplex panel for haplotype analysis and the triplex *SMN* PCR–minisequencing for direct *SMN1*-deletion analysis in whole-genome amplified single cells from GM03813, GM03814, and GM03815 parent–child trio. **(A)** Tridecaplex marker PCR results from an aliquot of the single-cell WGA product. **(B)** Triplex *SMN* PCR–minisequencing results from a second aliquot of the single-cell WGA product. **(C)** Haplotype phasing of the maternal and paternal wild-type and *SMN1*-deleted alleles. ADO, allele dropout, RFU, relative fluorescence unit; e8, exon 8; i7, intron 7; e7, exon 7.

Tridecaplex marker panel genotyping of parental cell lines GM03814 and GM03815 identified two fully informative markers *D5S610* and *SMA7115* and eight partially informative markers (*D5S1413*, *SMA6863*, *SMA6873*, *D5S1370*, *D5S1408*, *SMA7093*, *SMA7120*, and *D5S637*), while the remaining three markers were uninformative. Co-amplification of all 13 flanking microsatellite markers was observed from the WGA product of 21 out of 23 cells (**Figure 3**), with individual marker amplification failure of *SMA6877* observed in one cell, and amplification failures of *D5S1413* and *SMA6873* observed in another cell. Eleven of the 13 panel markers were heterozygous in at least one member of this trio and thus informative for ADO determination. Among the 21 cells with successful amplification of all markers, 12 cells showed no evidence of ADO for any of the 11 heterozygous markers, while ADO of between 1 and 3 individual markers was observed in the remaining 9 cells. There was no observable ADO for 5 of the 11 heterozygous markers. For the remaining six heterozygous markers, ADO rates ranged from 4.35% to 40%, which was similar to those observed in the above two unrelated cell lines (data not shown). The highest ADO rate was observed for *SMA6863*, which is also the largest amplicon in the panel. Nevertheless, it was possible to determine each cell’s diplotype correctly even for cells with ADO of one or more markers, because sufficient informative markers without ADO were available to establish haplotype phase.

Triplex PCR of *SMN* fragments e7, i7, and e8 was successful from the WGA product of all 23 cells. Triplex minisequencing detected only *SMN2*-specific e7, i7, and e8 nucleotides from all GM03813 cells (100% specificity), consistent with homozygous deletion of *SMN1* in this cell line derived from an SMA-affected child (**Figure 3**).

*SMN1*- and *SMN2*-specific e7, i7, and e8 amplicons were detected in all GM03815 cells and in five GM03814 cells. In the remaining three GM03814 cells, *SMN2*-specific e7, i7, and e8 amplicons were all clearly detected, but not all the *SMN1*-specific amplicons were observed. Only the *SMN1*-specific e7 and e8 amplicons were observed for one cell, and only the *SMN1*-specific e8 amplicon was observed for the other two cells. The dropout of one or two *SMN1*-specific amplicons in these cells is likely related to this cell line’s significant 1:5 ratio of *SMN1* to *SMN2* gene copies (Stabley et al., 2015), which would be expected to translate to poorer amplification yield of *SMN1*-specific amplicons. Nonetheless, the correct diagnosis was made on these three cells, since at least one of the three *SMN1*-specific amplicons was detected successfully. The independent amplification of the e7, i7, and e8 amplicons therefore provides triple redundancy for direct detection of *SMN1* presence/absence, while at the same time demonstrating robustness in detecting single-copy *SMN1* even in the presence of a fivefold excess of *SMN2*.

**Figure 4:**
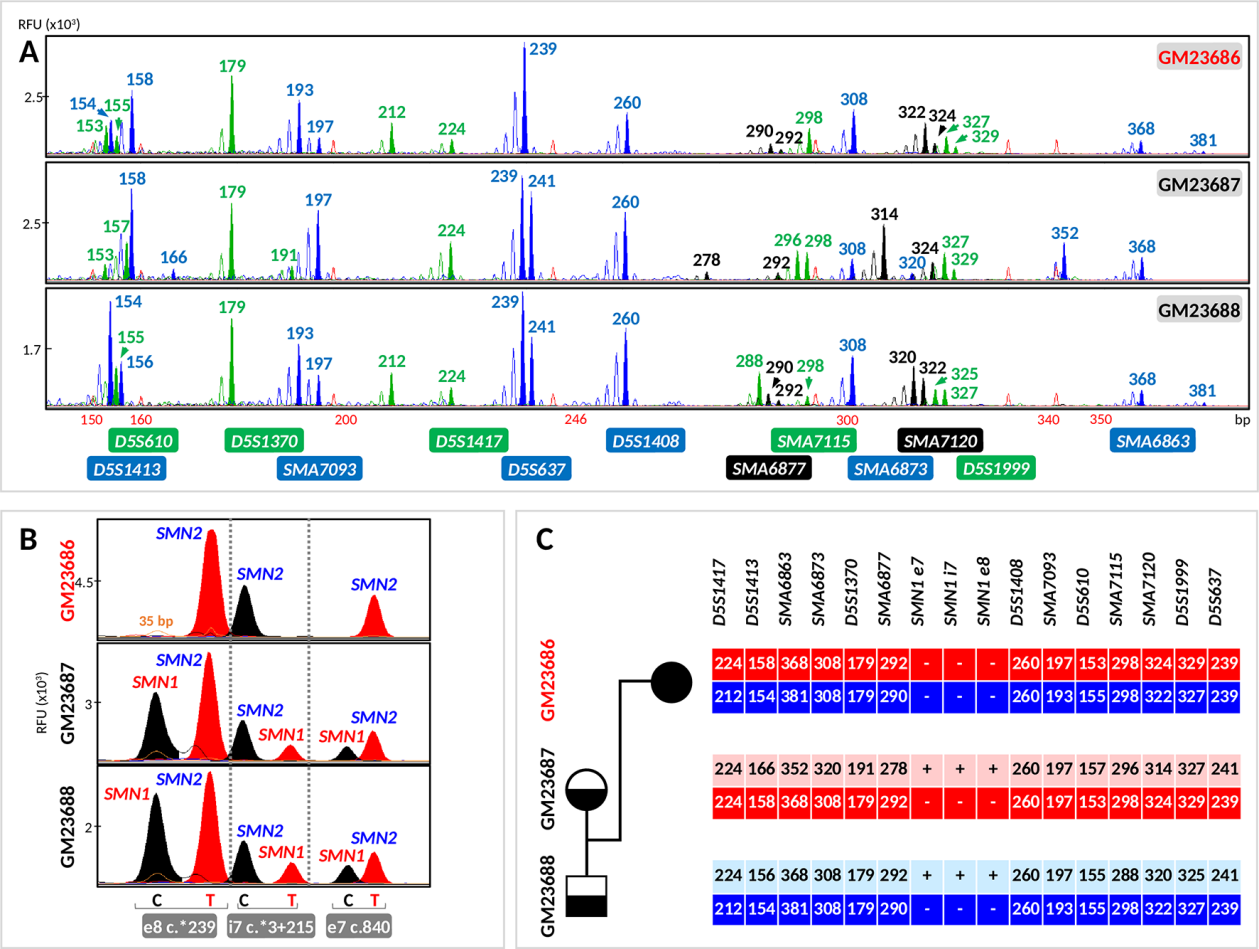
Evaluation of the tridecaplex panel for haplotype analysis and the triplex *SMN* PCR–minisequencing for direct *SMN1*-deletion analysis in whole-genome amplified single cells from GM23686, GM23687, and GM23688 parent–child trio. **(A)** Tridecaplex marker PCR results from an aliquot of the single-cell WGA product. **(B)** Triplex *SMN* PCR–minisequencing results from a second aliquot of the single-cell WGA product. **(C)** Haplotype phasing of the maternal and paternal wild-type and *SMN1*-deleted alleles. RFU, relative fluorescence unit; e8, exon 8; i7, intron 7; e7, exon 7.

We performed further validation on the second trio using 10 replicate single cells of each cell line. Of the 30 cells, 2 cells from GM23686 and 1 cell from GM23688 did not show any amplification and hence were excluded from further analysis. Two markers were identified as fully informative (*D5S1413* and *SMA7120*), nine were partially informative (*D5S1417*, *SMA6863*, *SMA6873*, *D5S1370*, *SMA6877*, *SMA7093*, *D5S610*, *SMA7115*, and *D5S1999*), and the remaining two were uninformative (**Figure 4**). Co-amplification of all markers was observed in 25 cells, while amplification failure of *D5S1413*, *SMA6863*, and *SMA6873* was observed in 1 cell and amplification failure of *SMA6877*, *SMA7115*, and *SMA7120* was observed in another cell. In this trio, 12 of the 13 markers were heterozygous in at least one member and thus informative for ADO determination. Among the 25 cells with successful amplification of all markers, 14 cells showed no evidence of ADO for any of the 12 heterozygous markers, while ADO of between 1 and 5 individual markers was observed in the remaining 11 cells. There was no observable ADO for 2 of the 12 heterozygous markers. For the remaining 10 heterozygous markers, ADO rates ranged from 5.56% to 23.53%.

Triplex PCR of *SMN* fragments e7, i7, and e8 was successful from the WGA product of all 27 cells, with detection of both *SMN1*- and *SMN2*-specific amplicons in 8 (80%) GM23687 cells and in 7 (77.78%) GM23688 cells. Triplex minisequencing detected only *SMN2*-specific e7, i7, and e8 nucleotides from all GM23686 cells (100% specificity), consistent with homozygous deletion of *SMN1* in this cell line (**Figure 4**). *SMN1*-specific amplicons were absent in two cells each from GM23687 and GM23688, but haplotype results of the four cells clearly showed presence of the normal parental alleles, thus confirming the unaffected status of these cells (data not shown).

Haplotypes linked to the parental normal and mutant (*SMN1*-deleted) alleles were established by correlation analysis of the triplex *SMN* PCR–minisequencing results with the tridecaplex PCR marker genotypes (**Figures 3**, **4C**). No discordance was observed between the presence/absence of *SMN1* and the haplotype assignments.

### Clinical IVF PGT-M of SMA

**Figure 5:**
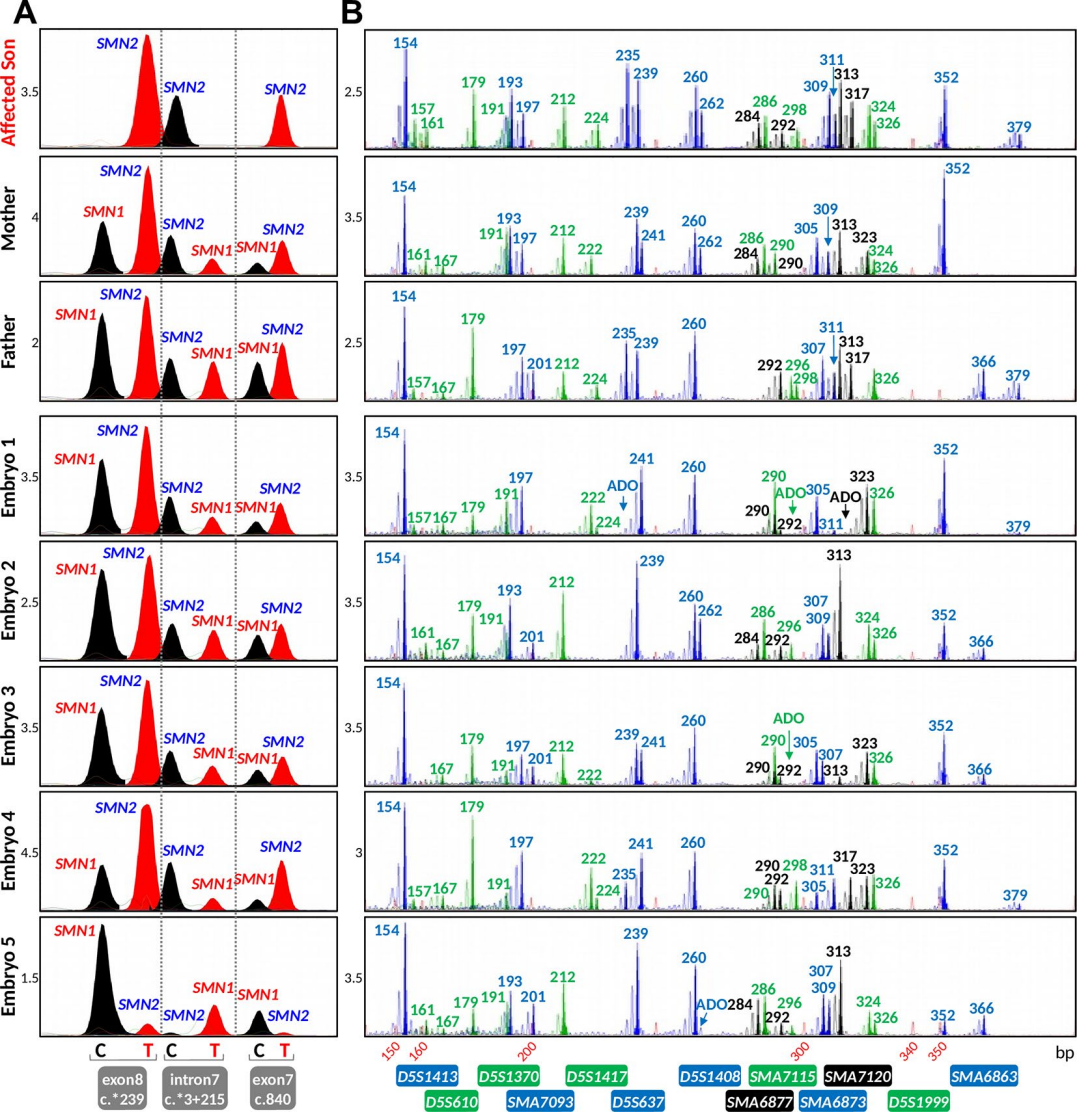
Results of the spinal muscular atrophy (SMA) IVF preimplantation genetic testing for monogenic disorder (PGT-M) cycle. **(A)** Triplex *SMN* PCR–minisequencing results. **(B)** Tridecaplex marker PCR results. The results representing the affected son and carrier parents were generated from genomic DNA, whereas those representing the embryos were derived from whole-genome amplified product of single blastomeres. ADO, allele dropout.

**Figure 6:**
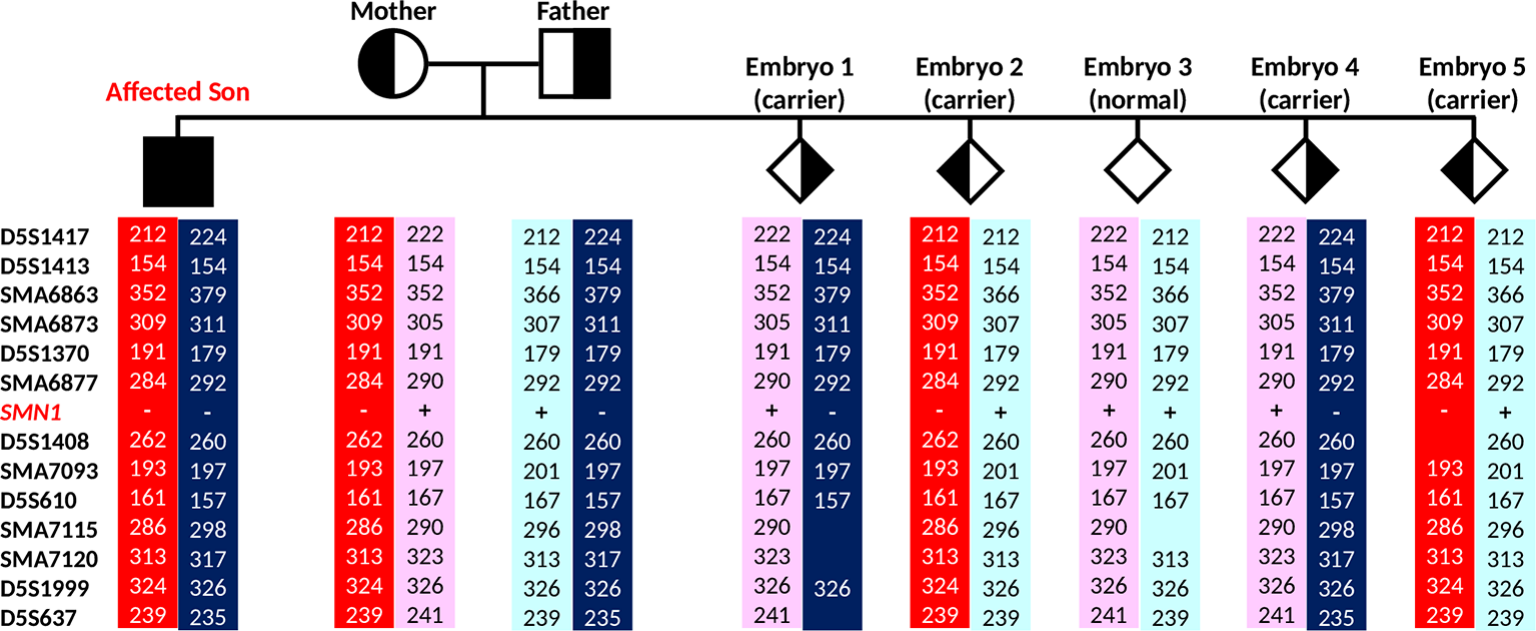
Marker diplotypes of five embryos from the PGT-M cycle. The parental mutant chromosomes are highlighted in red (maternal) and dark blue (paternal). Carrier and unaffected embryos are indicated by semi-filled and empty diamond symbols.

The husband was previously genotyped as carrying one copy each of *SMN1* and *SMN2*, while the wife carries one copy of *SMN1* and two copies of *SMN2*. Triplex *SMN* fragment (e7, i7, and e8) PCR and minisequencing confirmed the homozygous absence of *SMN1* in the affected son (**Figure 5**). Tridecaplex marker genotyping of the parents and affected son was performed (**Figure 5**) to establish the haplotypes of the maternal and paternal mutant (*SMN1*-deleted) and normal alleles (**Figure 6**).

In the IVF PGT-M cycle, five of nine oocytes fertilized after ICSI, and all five resultant embryos were biopsied on day 3 post-fertilization. Since the parental mutant/*SMN1*-deleted and normal/*SMN1*-positive haplotypes had been established *a priori* with the aid of the affected son’s DNA, stand-alone haplotype phasing would have sufficed for PGT-M (**Figure 6**). However, since this was the first time that the tridecaplex marker panel was used in PGT-M of SMA, direct *SMN1* deletion analysis was performed in parallel to evaluate the concordance between the two assays’ results when performed on single blastomeres. The SMA PGT-M cycle is summarized in **Table 3**.

**Table 3:** Outcome of IVF PGT-M for SMA.

Oocytes recovered	9
Oocytes fertilized with two pronuclei	5
Embryos biopsied	5
Unaffected embryos	1
Embryos transferred during the same cycle	1
Affected embryos	0
Carrier embryos	4
Embryos transferred during the same cycle	0
Embryos frozen	3
Frozen thawed embryos transferred at a subsequent cycle	1
Positive hCG	1
Pregnancy with fetal heartbeat	1
Live birth	1

PGT-M, preimplantation genetic testing for monogenic disorder; SMA, spinal muscular atrophy; hCG, human chorionic gonadotropin.

Triplex *SMN* amplicon PCR and minisequencing revealed that none of the five embryos had homozygous deletion of *SMN1*, but could not distinguish between unaffected and carrier embryos. In contrast, multi-marker haplotype analysis not only determined that none of the embryos was affected, but also established that one embryo was unaffected while the remaining four were heterozygous carriers. Same cycle transfer of the unaffected embryo did not lead to implantation, but subsequent frozen-thaw transfer of a single carrier embryo produced a pregnancy. An amniocentesis was performed which confirmed the PGT-M genotyping results. A live birth was subsequently delivered at term.

## Discussion

PGT-M of SMA presents certain challenges due to the fact that the critical *SMN1* gene lies within a tandemly duplicated chromosomal region of ∼1.7 Mb, differing from the nearly identical *SMN2* within this duplicon at only five nucleotide positions. Stand-alone direct detection of *SMN1* presence is susceptible to ADO or exogenous DNA contamination and the resultant misdiagnosis; therefore, complementation with indirect linkage analysis of flanking markers can increase diagnostic confidence. A multi-marker panel further reduces misdiagnosis, as haplotype assignment is highly tolerant of random ADO of individual markers when there is ample marker redundancy. It would also be highly unlikely for any exogenous DNA contamination to go undetected in the presence of multiple polymorphic microsatellite markers.

We have identified all microsatellite markers lying within 0.5 Mb on either side of the chromosome 5q13.2 duplicated segment, and developed a single-tube tridecaplex PCR assay for simplified marker genotyping and haplotype-based PGT-M of SMA. As suggested from the population screening results, this multi-marker panel may be able to provide sufficient informativity and marker redundancy for most SMA PGT-M cases without the need to identify additional markers, which is both time-consuming and laborious. However, as marker heterozygosities may differ among different ethnic groups, further assessment may be required when using the panel for other ethnic groups. The upstream and downstream panel markers have a maximum theoretical probability of recombination with *SMN1* of <2% and <1%, respectively, which are much lower than many previously reported markers (Moutou et al., 2003; Moutou et al., 2006; Girardet et al., 2008; Burlet et al., 2010; Korzebor et al., 2013).

Assuming that an affected offspring is available for disease haplotype phasing, haplotype-based PGT-M also distinguishes between paternal and maternal wild-type and mutant chromosomes, thus enabling identification of normal and carrier embryos if desired. This represents another advantage over stand-alone mutation detection, where reliable *SMN1* and *SMN2* copy number determination from single cells using current quantitative methods such as multiplex ligation-dependent probe amplification (MLPA) and digital PCR (Anhuf et al., 2003; Huang et al., 2007; Stabley et al., 2015; Feng et al., 2017) remains elusive. As a result, the affected embryos can only be distinguished from the unaffected (carrier or normal) embryos by detecting the absence/presence of *SMN1*, but carriers cannot be distinguished from normals. In contrast, the tridecaplex marker panel’s ability to establish unique parental normal/*SMN1*-positive and mutant/*SMN1*-deleted haplotypes was successfully evaluated in whole-genome amplified single cells isolated from cell lines of a parent–child trio with previously determined *SMN1* and *SMN2* copy numbers. The results from this evaluation, as well as from the clinical IVF PGT-M case, indicate that this assay is robust even for single cells or day 3 single blastomeres after WGA. Therefore, even though ADO-mediated misdiagnosis is a greater concern in IVF PGT-M involving single blastomeres after WGA compared with day 5 multi-cell trophectoderm samples, it can be effectively overcome by using a sufficiently large panel of highly polymorphic markers. In the unlikely event that insufficient informative markers are identified in a couple after screening the tridecaplex marker panel, other markers identified in **Table S1** may serve as a resource of additional potentially informative markers.

As noted above, IVF PGT-M of SMA can rely solely on multi-marker haplotype analysis when DNA from an affected offspring is available for *a priori* assignment of the parental normal/*SMN1*-positive and mutant/*SMN1*-deleted haplotypes. In the absence of DNA from an affected offspring or other source of haplotype phasing, stand-alone haplotype analysis is not possible. Hence, for at-risk couples in such situations, direct *SMN1* deletion analysis is necessary in order to identify affected (homozygous deleted) embryos. Performing direct *SMN1* deletion analysis in parallel with multi-marker genotyping analysis is a better option, as it allows exogenous DNA contamination to be easily detected, when present. Furthermore, if no contamination is detected, the parental haplotype phases can be assigned during the actual PGT-M, as long as there is one affected embryo, i.e. one that is homozygous deleted for *SMN1*. Once normal/*SMN1*-positive and mutant/*SMN1*-deleted haplotype phases have been assigned, it would be possible to identify the unaffected, carrier, and affected embryos.

## Data Availability Statement

All datasets generated for this study are included in the article/**Supplementary Files**.

## Author Contributions

SC conceptualized and coordinated the project and experimental design and revised the manuscript. MZ conducted the comparison experiments, data analysis, and interpretation, and wrote the manuscript. ML, FC, and AT performed the PGT analysis and verified the experimental and diagnostic test results. AA coordinated patient care, IVF/ICSI, and embryo transfer procedures and reviewed and approved the manuscript.

## Funding

This study was supported by a grant from Khoo Teck Puat—National University Children’s Medical Institute, National University Health System, Singapore (N-178-000-063-001).

## Conflict of Interest

The authors declare that the research was conducted in the absence of any commercial or financial relationships that could be construed as a potential conflict of interest.
