# Supplementary figures and images for: Identification of Novel Microsatellite Markers Flanking the SMN1 and SMN2 Duplicated Region and Inclusion Into a Single-Tube Tridecaplex Panel for Haplotype-Based Preimplantation Genetic Testing of Spinal Muscular Atrophy
Source: Front Genet. 2019 Nov 6;10:1105. doi: 10.3389/fgene.2019.01105 (PMC6851269; doi:10.3389/fgene.2019.01105)

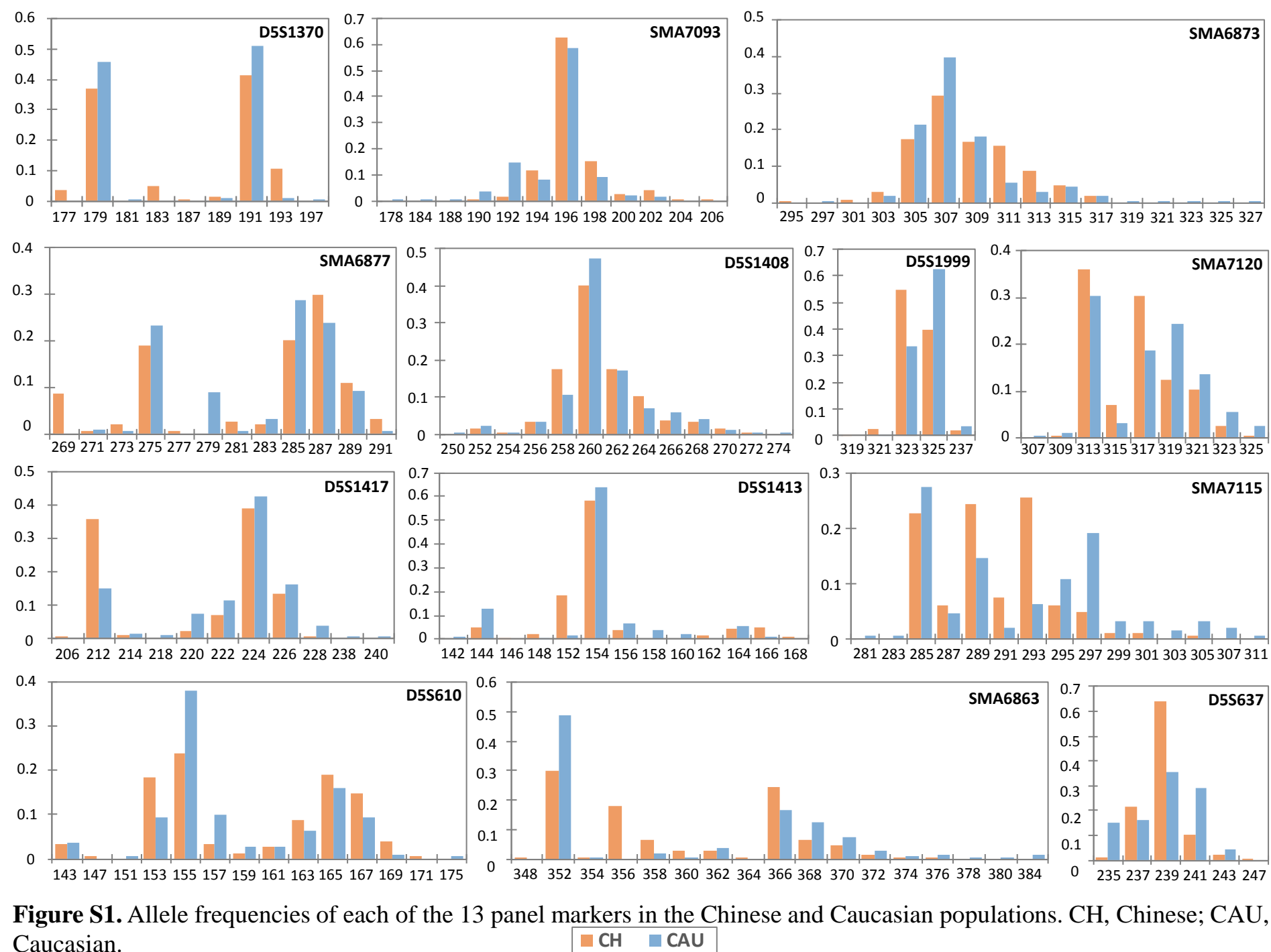

Supplement: Supplementary file 1 [file Image_1.pdf]
