# Supplementary material for: Identification of Novel Microsatellite Markers Flanking the SMN1 and SMN2 Duplicated Region and Inclusion Into a Single-Tube Tridecaplex Panel for Haplotype-Based Preimplantation Genetic Testing of Spinal Muscular Atrophy
Source: Front Genet. 2019 Nov 6;10:1105. doi: 10.3389/fgene.2019.01105 (PMC6851269; doi:10.3389/fgene.2019.01105)
Supplement: Supplementary file 2 [file Table_1.pdf]

Table S1. Microsatellite markers flanking the *SMN1* and *SMN2* duplicated region on chromosome 5q13.2, identified using Tandem Repeat Finder (TRF)

*Name**	*Location†*	*Repeat motif*	*No. of repeats*	*Percentage of matches‡*	*Score¶*
SMA6826	68263756-68264756	AGGGA	27.8	91	196
SMA6827	68278024-68278059	AATT	9	100	72
D5S629	68295567-68295602	TG	18	100	72
D5S1417	68307739-68307773	TG	17.5	100	70
SMA6831	68317321-68318321	AGGGG	8.8	100	88
SMA6832	68328800-68329800	ACAAA	5.2	100	52
SMA6835	68351286-68351363	AAAG	18.8	87	86
SMA6836	68362515-68362541	AC	13.5	100	54
SMA6838	68387476-68387524	TG	24.5	100	98
SMA6843.1	68430403-68431403	AAAAT	5.8	100	58
SMA6843.2	68433651-68434651	TTTTG	5.8	100	58
SMA6843.3	68435332-68436332	CTTTT	7	93	61
SMA6843	68439947-68439973	TA	13.5	100	54
D5S1364	68447815-68447841	CT	13.5	100	54
SMA6845	68459855-68459884	AT	15	100	60
SMA6847	68471802-68471843	TA	21.5	92	68
D5S1413	68514299-68514327	GT	14.5	100	58
SMA6855	68557319-68557368	TG	25.5	91	84
SMA6858	68582017-68582062	TA	23	100	92
SMA6861	68616615-68616650	TG	18	88	54
SMA6863	68630622-68630677	GA	28	88	85
SMA6865	68650860-68651860	AAAAC	7.4	90	58
SMA6870	68706794-68706825	TA	16	100	64
SMA6873	68732231-68732281	AC	25.5	100	102
SMA6874.1	68742581-68743581	GGGAA	8.6	97	68
D5S1370	68745154-68745192	TG	19.5	100	78
SMA6874.2	68747843-68747921	AAGG	19.8	87	117
SMA6874.3	68749860-68749918	TTTA	14.8	96	100
SMA6877	68770726-68770760	TG	17.5	100	70
SMN2	69345350-69373418	-	-	-	-
SMN1	70220768-70248838	-	-	-	-
D5S1408	70746933-70746985	AC	26.5	100	106
SMA7077	70774859-70774887	TTTTA	5.8	100	58
SMA7078	70787535-70787562	TTTTG	5.6	100	56
SMA7080	70802615-70802652	AC	19	100	76
SMA7091	70914303-70914379	TTCC	19.5	94	138
SMA7092	70928920-70928960	TA	20.5	100	82
SMA7093	70939862-70939899	TG	19	100	76

SMA7096.1	70961500-70961560	AAAAT	12.2	96	113
SMA7096.2	70963147-70963306	TTCC	41.5	81	102
SMA7097	70970774-70970910	TTTC	33.8	90	208
D5S610	70986953-70986987	TG	17.5	100	70
SMA7101.1	71011420-71011448	TTTTG	5.8	100	58
SMA7101.2	71017075-71017121	TG	24	86	69
SMA7103.1	71031924-71032105	TTTC	44.5	90	242
SMA7103.2	71034151-71034189	TTTA	9.8	100	78
SMA7104	71043866-71043916	AAAAT	9.8	87	75
SMA7114	71141935-71142020	TA	39.5	84	109
SMA7115	71158447-71158501	AG	28.5	85	78
SMA7117	71175458-71175486	AAAAC	5.8	100	58
SMA7119	71192642-71192670	AT	14.5	100	58
SMA7120	71204409-71204446	AC	19	100	76
D5S1999	71221597-71221648	GA	28	81	58
	71221654-71221696	CA	21.5	95	77

*Established markers are named as published. Novel markers contain the SMA prefix followed by their distance (in 10^4 base pairs) from chromosome 5pter, based on the UCSC genome browser reference sequence (GRCh37/hg19).
†Base pairs from chromosome 5pter, based on GRCh37/hg19.
‡Overall percentage of matches between adjacent copies as calculated by TRF (http://tandem.bu.edu/trf/trf.html).
¶Alignment score as calculated by TRF with settings as +2 for a base pair match and -7 for a mismatch or an indel in the repeat stretch. Alignment score is the weight for match, mismatch and indels.
Grey shaded markers are located within *Alu* sequences; markers in grey letters were difficult to design primers for; markers in **bold** letters are those selected for the multiplex-PCR panel.
