# Supplementary material for: Identification of Novel Microsatellite Markers Flanking the SMN1 and SMN2 Duplicated Region and Inclusion Into a Single-Tube Tridecaplex Panel for Haplotype-Based Preimplantation Genetic Testing of Spinal Muscular Atrophy
Source: Front Genet. 2019 Nov 6;10:1105. doi: 10.3389/fgene.2019.01105 (PMC6851269; doi:10.3389/fgene.2019.01105)
Supplement: Supplementary file 3 [file Table_2.pdf]

Table S2. Allele frequency distribution of microsatellite markers flanking the *SMN1* and *SMN2* duplicated region

Allele No.	D5S1417			D5S1413			SMA6863			SMA6873			D5S1370		
	Allele	Frequency		Allele	Frequency		Allele	Frequency		Allele	Frequency		Allele	Frequency	
		CH	CAU		CH	CAU		CH	CAU		CH	CAU		CH	CAU
1	206	0.01	-	143	-	0.01	348	0.01	-	295	0.01	-	177	0.04	-
2	212	0.36	0.16	145	0.05	0.13	352	0.30	0.49	297	-	0.01	179	0.37	0.46
3	214	0.01	0.02	147	0.01	-	354	0.01	0.01	301	0.01	-	181	-	0.01
4	218	-	0.01	149	0.02	0.01	356	0.18	-	303	0.03	0.02	183	0.05	-
5	220	0.02	0.07	151	0.18	0.02	358	0.07	0.02	305	0.17	0.21	187	0.01	-
6	222	0.07	0.12	153	0.58	0.64	360	0.03	0.01	307	0.29	0.40	189	0.02	0.01
7	224	0.39	0.43	155	0.04	0.07	362	0.03	0.04	309	0.17	0.18	191	0.41	0.52
8	226	0.14	0.16	157	-	0.04	364	0.01	-	311	0.16	0.06	193	0.11	0.01
9	228	0.01	0.04	159	-	0.02	366	0.24	0.17	313	0.09	0.03			
10				161	0.02	-	368	0.07	0.13	315	0.05	0.05			
11				163	0.04	0.06	370	0.05	0.07	317	0.02	0.02			
12				165	0.05	0.01	372	0.02	0.03	319	-	0.01			
13				167	0.01	-	374	0.01	0.01	321	-	0.01			
14							376	0.01	0.02	323	-	0.01			
15							378	-	0.01	325	-	0.01			
16							380	-	0.01	327	-	0.01			
17							384	-	0.02						
H_e		0.69	0.75		0.62	0.56		0.81	0.71		0.82	0.76		0.68	0.52
H_o		0.63	0.7		0.63	0.59		0.84	0.69		0.77	0.78		0.68	0.57

Allele No.	*SMA6877*			*D5S1408*			*SMA7093*			*D5S610*			*SMA7115*		
	Allele	Frequency		Allele	Frequency		Allele	Frequency		Allele	Frequency		Allele	Frequency	
		CH	CAU		CH	CAU		CH	CAU		CH	CAU		CH	CAU
1	269	0.09	-	246	0.01	-	178	-	0.01	143	0.03	0.04	281	-	0.01
2	271	0.01	0.01	250	-	0.01	184	-	0.01	147	0.01	-	283	-	0.01
3	273	0.02	0.01	252	0.02	0.02	188	-	0.01	151	-	0.01	285	0.23	0.28
4	275	0.19	0.23	254	0.01	0.01	190	0.01	0.04	153	0.18	0.08	287	0.06	0.05
5	277	0.01	-	256	0.03	0.03	192	0.02	0.15	155	0.24	0.38	289	0.24	0.15
6	279	-	0.09	258	0.17	0.10	194	0.12	0.08	157	0.03	0.10	291	0.08	0.02
7	281	0.03	0.01	260	0.40	0.47	196	0.63	0.59	159	0.01	0.03	293	0.26	0.06
8	283	0.02	0.03	262	0.17	0.17	198	0.15	0.09	161	0.03	0.03	295	0.06	0.11
9	285	0.20	0.29	264	0.10	0.07	200	0.03	0.02	163	0.09	0.06	297	0.05	0.19
10	287	0.30	0.24	266	0.04	0.06	202	0.04	0.02	165	0.19	0.16	299	0.01	0.03
11	289	0.11	0.09	268	0.03	0.04	204	0.01	-	167	0.15	0.10	301	0.01	0.03
12	291	0.03	0.01	270	0.02	0.01	206	0.01	-	169	0.04	0.01	303	-	0.02
13				272	0.01	0.01				171	0.01	-	305	0.01	0.03

14				274	-	0.01				175	-	0.01	307	-	0.02
15													311	-	0.01
H_e		0.81	0.79		0.76	0.72		0.57	0.61		0.84	0.80		0.81	0.84
H_o		0.83	0.8		0.79	0.7		0.57	0.56		0.80	0.80		0.85	0.81

**Allele No.**	***SMA7120***			***D5S1999***			***D5S637***		
	**Allele**	**Frequency**		**Allele**	**Frequency**		**Allele**	**Frequency**	
		**CH**	**CAU**		**CH**	**CAU**		**CH**	**CAU**
1	307	-	0.01	319	0.01	-	235	0.01	0.15
2	309	0.01	0.01	320	0.02	-	237	0.22	0.16
3	313	0.36	0.30	321	0.01	0.01	239	0.65	0.35
4	315	0.07	0.03	322	0.08	0.03	241	0.10	0.29
5	317	0.30	0.19	323	0.46	0.30	243	0.02	0.04
6	319	0.13	0.24	324	0.01	-			
7	321	0.10	0.14	325	0.40	0.63			
8	323	0.03	0.06	327	0.02	0.04			
9	325	0.01	0.03						
H_e		0.75	0.79		0.62	0.52		0.52	0.74
H_o		0.75	0.75		0.62	0.6		0.54	0.86

CH, Chinese; CAU, Caucasian.
